# Supplementary material for: The INSIDEOUT framework provides precise signatures of the balance of intrinsic and extrinsic dynamics in brain states
Source: Commun Biol. 2022 Jun 10;5:572. doi: 10.1038/s42003-022-03505-7 (PMC9187708; doi:10.1038/s42003-022-03505-7)
Supplement: Supplementary file 2 — Supplementary Material [file 42003_2022_3505_MOESM2_ESM.pdf]

Supplementary material

Inside out: Precise signatures of the balance of intrinsic and extrinsic dynamics in brain states

Gustavo Deco, Yonatan Sanz Perl, Hernan Bocaccio, Enzo Tagliazucchi and Morten L. Kringelbach

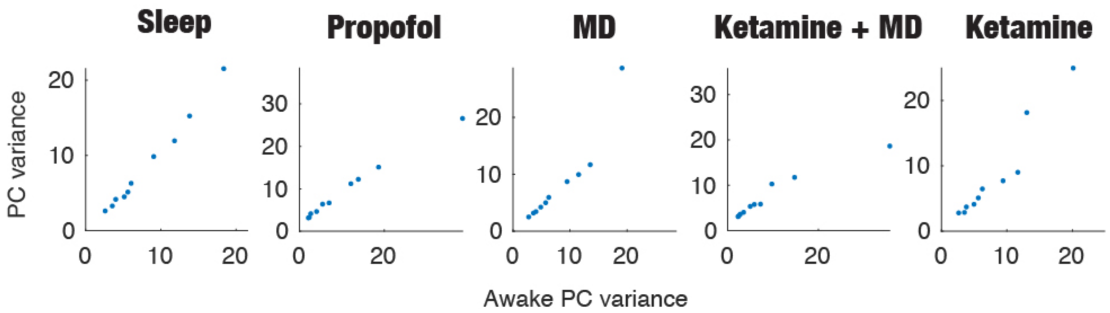

Supplementary Figure 1. Scatterplots of the PC variances which does not distinguish between conditions. *For each condition, the figure is showing the diagonal elements of the functional connectivity in PCA space with N=10 components as the INSIDEOUT framework, which, as can be seen, is not useful for distinguishing brain states.*

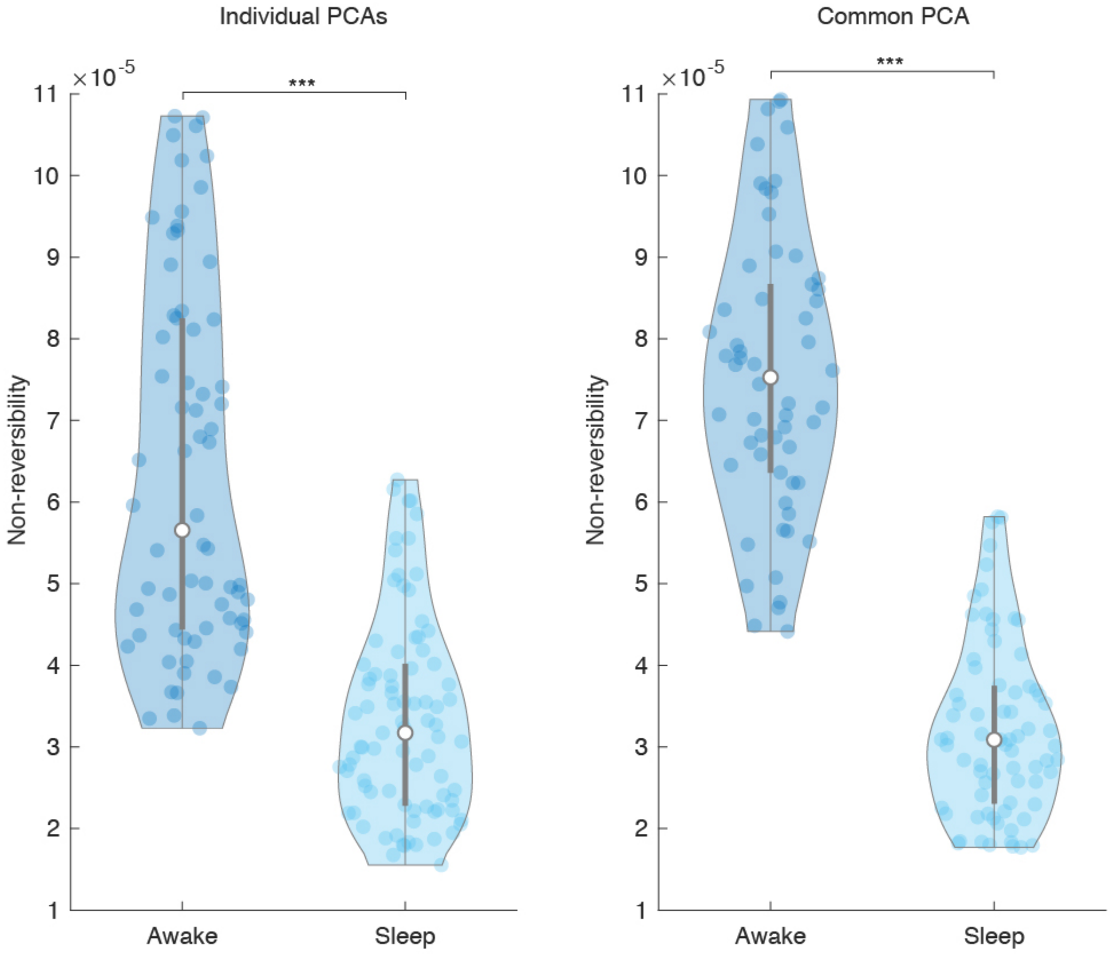

Supplementary Figure 2. Comparing strategies of using individual vs common reference PCAs. *The left panel of the figure shows the results of concatenating all the sessions for one monkey (Chibi) in awake and in sleep. The right panel shows the same using a common reference strategy. As can be seen, both strategies result in a similar level of significant differences in reversibility.*

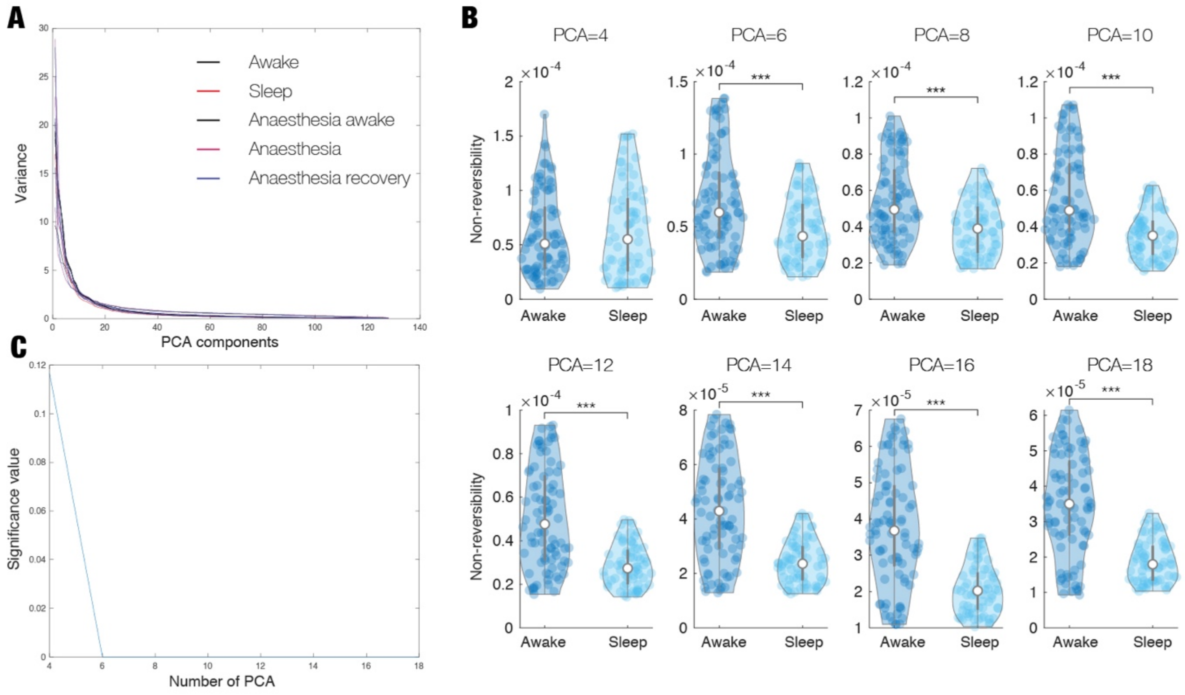

Supplementary Figure 3. The influence of the number of PCA components. ***A)*** *The panel is showing the variance of each PCA component for one monkey (Chibi) for all conditions, which are clearly overlapping. For N=10, this explains over 90% of the variance.* ***B)*** *The violinplots show the level of non-reversibility for awake and sleep in the same monkey for all even numbered PCAs from [4..18]. Except for N=4, all show a significant difference in non-reversibility between the two conditions.* ***C)*** *The panel shows the p-values in* ***B)****.*

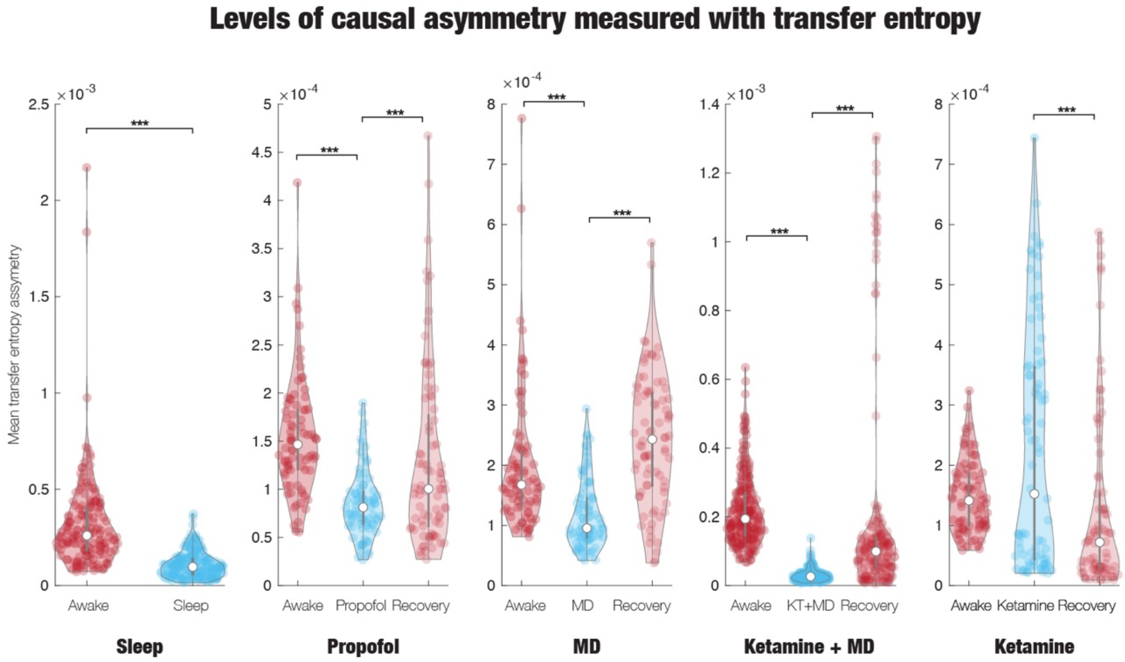

Supplementary Figure 4. Causal interactions between brain states. *We used the normalised directed transfer entropy (NDTE), an information-based measure of Granger causality, to compute the causal interactions between different regions using transfer entropy. The figure shows the results of comparing the five conditions in terms of comparing the levels of asymmetry, measured as the quadratic differences between the transfer entropy matrices (flow between pair of regions) and their transposed (see Methods). This level of asymmetry is a proxy for the breaking of the detailed balance. As can be seen from the figure, the NDTE results are consistent with those found with the INSIDEOUT framework and thus validates this. They also strengthen the interpretation of the link between non-reversibility/non-equilibrium and breaking the detailed balance.*

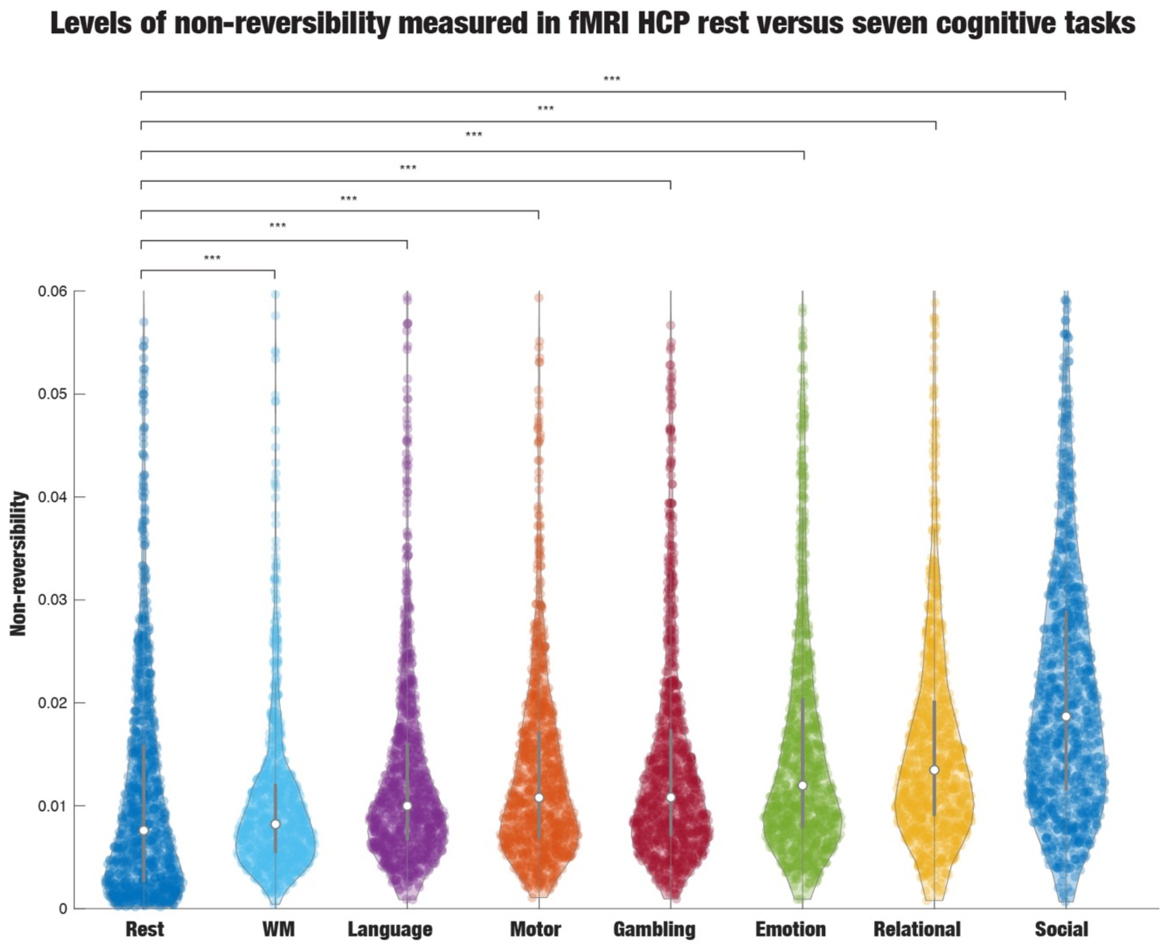

Supplementary Figure 5. Validating the INSIDEOUT framework with large-scale human HCP functional MRI neuroimaging data. *As can be seen from the violinplots for rest and seven cognitive tasks in over 1000 human participants, the results show that the level in non-reversibility increases in the seven tasks (covering the full cognitive domain) compared to rest.*

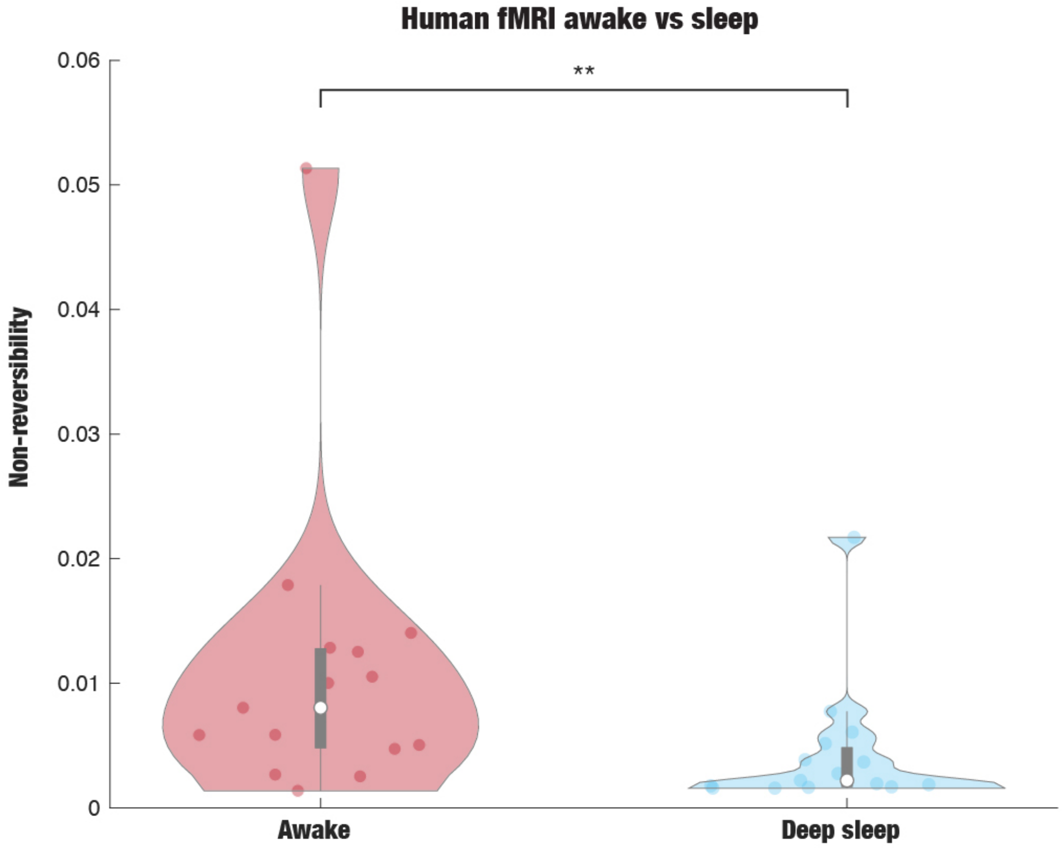

Supplementary Figure 6. Validating the INSIDEOUT framework in human fMRI data from different brain states. *The figure shows significant differences between wakefulness and deep sleep brain states in human fMRI.*
