# Supplementary material for: The INSIDEOUT framework provides precise signatures of the balance of intrinsic and extrinsic dynamics in brain states
Source: Commun Biol. 2022 Jun 10;5:572. doi: 10.1038/s42003-022-03505-7 (PMC9187708; doi:10.1038/s42003-022-03505-7)
Supplement: Supplementary file 3 — Description of Additional Supplementary Files [file 42003_2022_3505_MOESM3_ESM.pdf]

Description of Additional Supplementary Files

File name: Supplementary Data 1
Description: Datapoints for the violinplots in Figures 3C and 4.
