# Supplementary material for: The INSIDEOUT framework provides precise signatures of the balance of intrinsic and extrinsic dynamics in brain states
Source: Commun Biol. 2022 Jun 10;5:572. doi: 10.1038/s42003-022-03505-7 (PMC9187708; doi:10.1038/s42003-022-03505-7)
Supplement: Supplementary file 5 — Reporting Summary [file 42003_2022_3505_MOESM5_ESM.pdf]

nature portfolio

Corresponding author(s): Kringelbach, Deco

Last updated by author(s): 14-04-2022

Reporting Summary

Nature Portfolio wishes to improve the reproducibility of the work that we publish. This form provides structure for consistency and transparency in reporting. For further information on Nature Portfolio policies, see our Editorial Policies and the Editorial Policy Checklist.

Statistics

For all statistical analyses, confirm that the following items are present in the figure legend, table legend, main text, or Methods section.

n/a	Confirmed	
☐	☒	The exact sample size (n) for each experimental group/condition, given as a discrete number and unit of measurement
☐	☒	A statement on whether measurements were taken from distinct samples or whether the same sample was measured repeatedly
☐	☒	The statistical test(s) used AND whether they are one- or two-sided<br>*Only common tests should be described solely by name; describe more complex techniques in the Methods section.*
☐	☒	A description of all covariates tested
☐	☒	A description of any assumptions or corrections, such as tests of normality and adjustment for multiple comparisons
☐	☒	A full description of the statistical parameters including central tendency (e.g. means) or other basic estimates (e.g. regression coefficient) AND variation (e.g. standard deviation) or associated estimates of uncertainty (e.g. confidence intervals)
☐	☒	For null hypothesis testing, the test statistic (e.g. F, t, r) with confidence intervals, effect sizes, degrees of freedom and P value noted<br>*Give P values as exact values whenever suitable.*
☒	☐	For Bayesian analysis, information on the choice of priors and Markov chain Monte Carlo settings
☒	☐	For hierarchical and complex designs, identification of the appropriate level for tests and full reporting of outcomes
☐	☒	Estimates of effect sizes (e.g. Cohen's d, Pearson's r), indicating how they were calculated

Our web collection on statistics for biologists contains articles on many of the points above.

Software and code

Policy information about availability of computer code

Data collection	ECOG collection, public domain data from non-human primates, described on http://wiki.neurotycho.org. Human neuroimaging data from from Human Connectome Project, while the human sleep data was provided by Dr Helmut Laufs.
Data analysis	Custom-made MATLAB scripts were used for INSIDEOUT paradigm, which are freely available

For manuscripts utilizing custom algorithms or software that are central to the research but not yet described in published literature, software must be made available to editors and reviewers. We strongly encourage code deposition in a community repository (e.g. GitHub). See the Nature Portfolio guidelines for submitting code & software for further information.

Data

Policy information about availability of data

All manuscripts must include a data availability statement. This statement should provide the following information, where applicable:

- Accession codes, unique identifiers, or web links for publicly available datasets
- A description of any restrictions on data availability
- For clinical datasets or third party data, please ensure that the statement adheres to our policy

The code is freely available from github. ECOG data from the experiment are available on http://wiki.neurotycho.org. HCP data is freely available from http://www.humanconnectome.org. The human sleep data was provided by Dr Helmut Laufs.

Field-specific reporting

Please select the one below that is the best fit for your research. If you are not sure, read the appropriate sections before making your selection.

☒ Life sciences ☐ Behavioural & social sciences ☐ Ecological, evolutionary & environmental sciences

For a reference copy of the document with all sections, see nature.com/documents/nr-reporting-summary-flat.pdf

Life sciences study design

All studies must disclose on these points even when the disclosure is negative.

Sample size	We used data of 4 non-human primates from the public available database from Neurotycho. For the supplementary material we used 1000 human participants from HCP and 18 participants from the human sleep project.
Data exclusions	N/A
Replication	N/A
Randomization	N/A
Blinding	N/A

Reporting for specific materials, systems and methods

We require information from authors about some types of materials, experimental systems and methods used in many studies. Here, indicate whether each material, system or method listed is relevant to your study. If you are not sure if a list item applies to your research, read the appropriate section before selecting a response.

Materials & experimental systems

n/a	Involved in the study	
☒	☐	Antibodies
☒	☐	Eukaryotic cell lines
☒	☐	Palaeontology and archaeology
☐	☒	Animals and other organisms
☐	☒	Human research participants
☒	☐	Clinical data
☒	☐	Dual use research of concern

Methods

n/a	Involved in the study	
☒	☐	ChIP-seq
☒	☐	Flow cytometry
☐	☒	MRI-based neuroimaging

Animals and other organisms

Policy information about studies involving animals; ARRIVE guidelines recommended for reporting animal research

Laboratory animals	Public domain data reused. As described in details in the original paper by Yanagawa et al 2013, overall care was managed by the Division of Research Resource Center at RIKEN Brain Science Institute, where the non-human primates were housed in a large individual enclosure with other animals visible in the room, and maintained on a 12:12-h light:dark cycle. The multi-dimensional recording technique used chronically implanted, customized multichannel ECoG electrode arrays (Unique Medical, Japan). The 128 electrodes array (made of 3-mm diameter platinum discs), was implanted in the subdural space in 4 adult macaque monkeys (George, Chibi, Su and Kin2). The array had an interelectrode distance of 5 mm and were implanted in the left hemisphere, continuously covering over the frontal, parietal, temporal, and occipital lobes (Figure 2B, the detailed methods are described in Nagasaka et al 2011).
Wild animals	N/A
Field-collected samples	N/A
Ethics oversight	RIKEN ethical committee (No. H24-2-203(4)) approved all experimental and surgical procedures

Note that full information on the approval of the study protocol must also be provided in the manuscript.

Human research participants

Policy information about studies involving human research participants

Population characteristics	We used data a group of 1003 participants from the public available database from the Human Connectome Project (HCP) from the WU-Minn HCP Consortium.
Recruitment	The data set used for this investigation was selected from the March 2017 public data release from the Human Connectome Project (HCP) where we chose the full sample
Ethics oversight	HCP

Note that full information on the approval of the study protocol must also be provided in the manuscript.

Magnetic resonance imaging

Experimental design

Design type	Resting state and task design
Design specifications	1003 HCP participants. The HCP website (http://www.humanconnectome.org/) provides the full details of participants, the acquisition and preprocessing of the data.
Behavioral performance measures	N/A

Acquisition

Imaging type(s)	functional and diffusion MRI
Field strength	3T
Sequence & imaging parameters	The 1003 HCP participants were scanned on a 3-T connectome-Skyra scanner (Siemens). The HCP website (http://www.humanconnectome.org/) provides the full details of participants, the acquisition and preprocessing of the data.
Area of acquisition	Whole brain

Diffusion MRI ☐ Used ☒ Not used

Preprocessing

Preprocessing software	Standardized methods using FSL (FMRIB Software Library), FreeSurfer, and the Connectome Workbench software
Normalization	FLIRT
Normalization template	CIFTI
Noise and artifact removal	The HCP website (http://www.humanconnectome.org/) provides the full details of the preprocessing of the data.
Volume censoring	As per HCP pipeline

Statistical modeling & inference

Model type and settings	We used the INSIDEOUT framework to analyse the empirical data
Effect(s) tested	Spatiotemporal structure

Specify type of analysis: ☒ Whole brain ☐ ROI-based ☐ Both

Statistic type for inference (See Eklund et al. 2016)	N/A
Correction	N/A

Models & analysis

n/a	Involved in the study
☐	☒ Functional and/or effective connectivity
☒	☐ Graph analysis
☒	☐ Multivariate modeling or predictive analysis

Functional and/or effective connectivity	Pearson correlation
